# The Role of Autosensitivity Control (ASC) in Cochlear Implant Recipients

**DOI:** 10.3390/audiolres11010003

**Published:** 2021-01-21

**Authors:** Federica Di Berardino, Diego Zanetti, Daniela Soi, Lara Dalla Costa, Sandro Burdo

**Affiliations:** 1 Department of Specialist Surgical Sciences, Audiology Unit, Fondazione IRCCS Ca’ Granda Ospedale Maggiore Policlinico, 20122 Milan, Italy; federica.diberardino@unimi.it; 2 Department of Clinical Sciences and Community Health, University of Milan, 20122 Milano, Italy; 3 Italian Association “Liberi di Sentire”, formerly Director (SB) and Assistant (LDC) at Audiovestibology Unit Varese Hospital, 21100 Varese, Italy; danisoi@yahoo.it (D.S.); laradallacosta@me.com (L.D.C.); sanburdo@tin.it (S.B.)

**Keywords:** ADRO, ASC, CI, cochlear implantation, word recognition score, speech in noise, fitting

## Abstract

The purpose of the study was to examine the subjective and objective potential advantage for speech understanding in noise achieved by cochlear implant (CI) recipients when using the autosensitivity control (ASC) input signal processing in combination with the adaptive dynamic range optimization (ADRO). Eighteen subjects (8 females, 10 males, mean age 17.7 ± 6.7) were enrolled in a prospective open blinded comparative study between the ASC + ADRO condition vs. the ADRO alone; 16 were sequential binaural and 2 were monoaural CI recipients. All patients had been wearing their CI for at least 3 years, had no additional disabilities, had an age-appropriate receptive and expressive language. Word recognition performances in noise (at signal-to-noise ratio +5 dB HL) were significantly better in the ADRO-alone condition than in the ADRO + ASC condition. (*p* = 0.03) These objective outcomes were in agreement with the subjective reports. No significant difference was found in quiet. Our results, apparently in contrast with other reports in the literature, suggest that the decision of adding the slow-acting automatic reduction in microphone sensitivity provided by ASC should be limited to selected CI recipients.

## 1. Introduction

Among the input signal pre-processing systems of cochlear implantations (CI), many authors have described the benefits of the autosensitivity control (ASC) [1,2,3,4,5,6]. The ASC was firstly introduced in the body-worn mini sound processor in 1989 by Cochlear Limited (Lane Cove, Australia) [7] and was designed to improve speech recognition by keeping the speech level in the comfortable loudness region in the presence of background noise [8]. It is a slow-acting linear compression pre-processing system that acts after the amplification performed by the speech-processor microphone; its purpose is to optimize the speech-to-noise ratio, based on the unmodulated noise input (“noise floor”) of the surrounding environment [9]. The ASC attack time is generally 2 second, and the release time is 8 second; its long release time is used to reduce distortion and/or maintain a comfortable listening level [10]. While the overall noise floor is continuously monitored by the slow dynamics of ASC [8], any transient and abrupt increase in noise is not controlled by a slow system that misses them completely [9]. As a result, every time an unexpectedly loud sound occurs in the environment, the user perceives an uncomfortably loud stimulation [11,12]. In order to reduce the effect of impulsive noises, another pre-processing automatic gain control (AGC) has been implemented, called AGC-input, working together with ASC in a system defined as “dual-loop AGC”. While ASC acts like a slow-acting AGC, the AGC-input is built to react quickly in response to sudden loud noise, by reducing the gain of high-amplitude inputs and avoiding distortion and peak clipping [13,14]. Modulation rates and depths are used to classify a signal as speech (i.e., 2–20 Hz) or noise.

**Figure 1:**
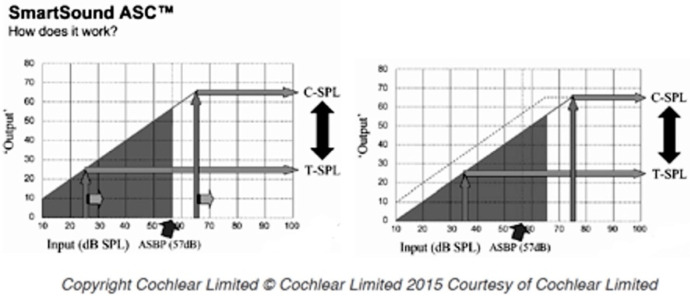
The autosensitivity control (ASC) input–output curves.

The ASC is activated when the average noise floor increases exceeding the automatic gain kneepoint, defined as the AutoSensitivity Break-Point (ASBP), that is, 57 dB SPL at default settings [15]. Once the ambient noise floor reaches or exceeds 57 dB SPL, whether it is impulsive or not, the sensitivity of the speech-processor microphone is slowly reduced by the ASC according to the level of the background noise; this produces a subjective reduction of loudness. In order to keep the noise floor at least 10–15 dB SPL below the AGC kneepoint [16], the ASC shifts all the instantaneous input dynamic range (IIDR), with a compression at a ratio of 1:1. Since the ASC acts as a linear amplifier with minimal variations in the signal, it reduces the whole input gain and not only the high intensities or the unmodulated sounds (Figure 1). This input signal pre-processing systems have been implemented in the Smartsound^®^ software by Cochlear (Cochlear Limited, Sydney, Australia); however, the literature on the real-world joint benefit of AGCs to ASC in CI recipients is still scarce, and some authors have raised the hypothesis that the ASC might negatively affect the interaural level difference cues [6]. In our clinical practice, we noticed that most of our patients frequently reported to prefer to use only the Adaptive Dynamic Range Optimization (ADRO) alone rather than ASC + ADRO in noisy conditions. The aim of this study was to examine the subjective and objective potential benefits or disadvantages in speech recognition in noise by the use of ASC input signal processing.

## 2. Materials and Methods

The study design is prospective, cross-sectional, observational blinded, focused on the direct effect of ASC vs. ADRO. As in the study conducted with children by Rakszawski B. et al. [17], the other preprocessing systems (Whisper^®^, Beam^®^ and Zoom^®^) (Cochlear Limited, Sydney, Australia) that modify the microphone directionality and adjust the electrode gain in noise were deactivated.

Eighteen CI recipients (8 females and 10 males, mean age of 17.7 ± 6.7 years, ranging from 10 to 46 years) were randomly selected to be enrolled in the study. All subjects had age-appropriate receptive and expressive language; none had additional disabilities. 

The demographics and the main clinical features of the patients are reported in Table 1. 

Participants had been implanted either with the Nucleus Freedom^®^ (Cochlear Limited, Sydney, Australia) or with a Nucleus 5 (CI512)^®^ (Cochlear Limited, Sydney, Australia). Sixteen were sequential binaural CI and two were monaural CI recipients. All patients used a CP810^®^ (Cochlear Limited, Sydney, Australia) speech processor. The mean duration of CI use was 9 years, with a range from 2 to 13 years. All participants used the Advanced Combination Encoder (ACE) speech coding strategy. 

All CI recipients’ maps were checked and re-programmed weekly for 6 to 8 weeks. Minimum stimulation (T) levels were set at counted thresholds to ensure audibility, and maximum stimulation (C) levels were set at loud but comfortable levels to ensure the dynamic range was maximized. All participants in this study were programmed according to this protocol after at least 3 years from their initial CI activation. All 22 electrode contacts were active in all CI recipients. A monopolar stimulation was selected for all patients, and stimuli were trains of symmetric biphasic pulses of 25 msec duration; the duration of each pulse-train was 600 msec. The rate of stimulation was 2400 pulses per second per channel (pps/ch) in 70% of patients and 1200 pps/ch in the other 30%.

All participants were fitted with two maps: one map included ADRO and the other map included ADRO + ASC; the patients were blinded to the allocation of the 2 maps and were asked to use arbitrarily the two maps in everyday life and in the different environment conditions (e.g., at home, in the pub, in the street traffic, at work). The sensitivity and volume of the microphone were kept at default settings. All subjects should indicate the map they considered most favourable and were interviewed regarding the subjective qualitative differences between the two maps. 

All subjects underwent a speech-tracking (ST) test in noise [18] by a trained audiologist in a double-blind setting, since both the patient and the Audiologist were unaware of which maps were tested. The ST takes four minutes to be accomplished; it is routinely used in the fitting protocol to evaluate the effectiveness of hearing aids and CI, given its high sensitivity in detecting any decline in hearing performance in intra-subject testing. It also investigates the recognition of ongoing speech, and it can be used as a method of training [19]. According to Burdo et al. [20], by counting the number of words repeated correctly in a minute, the ST returns a word recognition score (WRS) in a free-running connected discourse. 

The speech material consisted in 20 sentences extracted from common written texts chosen on the basis of the patient’s age; for adults, it consists of reading a newspaper (level +3), delivered live-voice by a professional reader (always the same one in all sessions with all patients), without lip-reading, in quiet and in noise, at a root mean square level of 65 dB (A) SPL, located 1 m directly in front of the proband (0° azimuth). The background (“cocktail party”) noise was delivered from a second loudspeaker located 1 m behind the patient’s shoulders (180° azimuth). Both signal and noise were adjusted in order to achieve a constant signal-to-noise (SNR) ratio of +5 dB HL. [21] The speech tests were performed in a sound-treated booth; live voice output was constantly monitored during the test, and the loudspeaker output was checked with a sound meter before each patient’s session.

Statistical analysis. The Kolmogorov–Smirnov test was used to assess the normality of the examined variables. The paired Student’s t-test was carried out to determine the statistical significance of average differences of scores obtained with the two maps. A *p*-value of less than 0.05 was considered statistically significant. All statistics were calculated using the Statistical Package for the Social Sciences 24.0 for Windows software package (SPSS Inc., Chicago, IL, USA).

## 3. Results

No association was found between the outcomes obtained with the two maps (with and without ASC) and the patients’ characteristics. WRS in quiet were not statistically different between ASC + ADRO compared to ADRO alone. 

**Figure 2:**
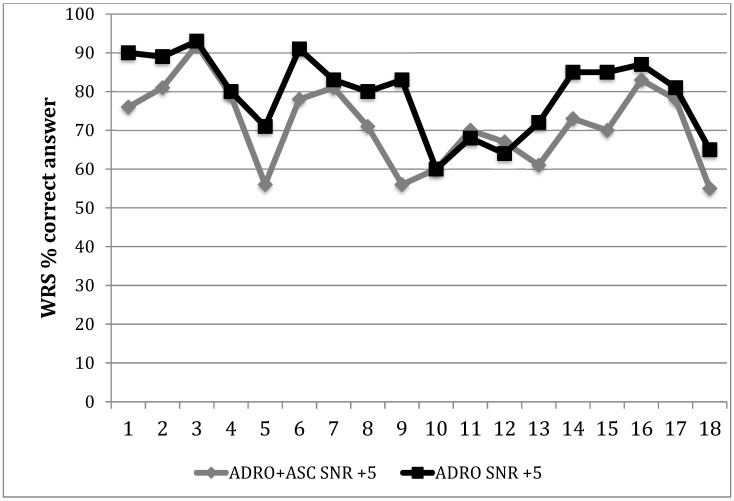
Word recognition scores for each CI recipient with the two settings.

Conversely, when the speech tracking task was performed with a masking noise, the difference between ADRO + ASC and ADRO alone was significant: 15 out of 18 patients (83.34%) scored significantly better at WRS with the ADRO alone, as shown in Figure 2, (*p* = 0.03) with an average improvement of 7 words per minute at a SNR of +5 dB HL. Patient 10 had the same WRS at SNR +5 dB HL with the two maps; only patient 11 and 12 had a slightly higher number of correct answers at WRS with ADRO + ASC compared with ADRO alone. However, they both indicated the ADRO as the preferred map, complaining about the reduction of speech recognition in noise with ASC, due to a subjective significant lowering of the speech signal intensity in noise.

From a subjective viewpoint, all patients reported discomfort when using the map with ADRO + ASC in quiet and noisy environments. Only two CI recipients reported to perceive sometimes a clearer voice with the ASC map in noise but, nevertheless, they preferred and used more frequently the ADRO alone. The major complaints with the ASC + ADRO map concerned the lowering of the loudness of speech and the perceived fluctuations of the voice, which resulted in a significant subjective reduction of speech comprehension in noise. On the contrary, all patients reported a certain improvement of the SNR in a noisy environment with ADRO alone.

## 4. Discussion

In the literature, the ASC has been consistently reported to provide a 2–3 dB SNR improvement of the 50% correct answers threshold either alone or in combination with ADRO [22]. In this current open, double-blinded investigation, we failed to observe the expected objective improvement in noise with ASC. Our results indicate that ADRO alone allows a significantly better response, with an average improvement of 7 words per minute at an SNR of +5 dB HL. The patients’ feedback was also in agreement with these objective results, since all the patients reported preferring the map with ADRO alone in different noisy environments. The major complaints reported by the patients when using ASC resemble the disadvantages of the slow-acting compression systems applied in hearing aids [23]:

- Loudness perception that is not restored to “normal”. The output level typically shifts only slightly from the input level; it may be difficult for the user to judge the strength of sound sources. This may have adverse effects on the interpretation of environmental sounds [24].
- When the acoustic scene changes abruptly, such as when two voices with markedly different levels alternate or when switching rapidly from a loud to a quiet environment (e.g., when leaving a noisy room), the gain takes a second or two to reach the value appropriate for the new situation. Hence, the aid may appear to become “dead” for a while.
- When trying to listen to one (target) voice in the presence of another (background) voice, a normally hearing person can extract information about the target during the temporal dips in the background, a process called “Listening in the dips” [25]. The information in the dips may be at a relatively low level, especially when the mean target level is lower than the mean background level. Hearing-impaired people have a reduced ability to listen in the dips [23], partly because of reduced audibility of the target speech in the dips [26]. A slow-acting system may be of limited benefit in this situation because the gain does not increase significantly during brief dips in the input signal; the gain applied during the dips is essentially the same as the gain applied during the peaks in the input.

Conversely, as already reported by James et al. in 2003 [27], the results in quiet observed in this study did not show any difference between the two maps. Therefore, we tried to understand why introducing the ASC did not bring in our data the expected objective improvement in noise.

First of all, according to the classification of the compression algorithms proposed by Dillon [12], the ASC has a linear slow-acting dynamics with a medium compression threshold. While fast-acting compression with a short release time ( <50 ms) such as the AGC-input component is designed to follow the intensity variations encountered at the phonemic or syllabic level of speech, longer release time (>200 ms), such as the ASC, is used to reduce distortion and/or maintain a comfortable listening level; as a result, the ASC is more useful when the change in gain is needed for larger intensity levels and longer duration. For this reason, the improvement observed with ASC may differ in the presence of other types of noise or environments (i.e., steady-state noise or diffuse noise in a reverberant environment) [2], and different levels of speech and noise influence the real-life outcomes. 

As an example, a child’s everyday listening environment is much noisier than that encountered by the typical adult [28]. However, we noticed that the school background noise reported in the ASC literature is much higher (dB Leq/day: 87.3 dBA to 95.5 dBA [29], with a mean of 56 dBA during silent classroom reading to 73 dB A during group activities [28]) than the noise level found in our schools, which are more similar but lower than other European reports (dB Leq/day 51.5 dBA ± 4.5 dBA; ranging from 38 to 58 dBA) [30]. This observation might confirm that the beneficial effect of ASC + ADRO is more evident in diagnostic and real-life settings in which there are higher levels of constant surrounding noise and higher root-mean-squared output level. 

As far as CIs are concerned, previous research demonstrated that loudness grows as an exponential function of the current intensity [31,32]. It is also known that the loudness growth functions are dependent on pulse rate, with loudness growing faster at low pulse rates [33,34]. Electrical thresholds and maximum acceptable loudness levels, in fact, vary for stimulation rates between 250 and 2400 pps/ch on the absolute current level (CL) value [35]. T and C levels decrease as a function of pulse rate but the slopes of the C level vs. pulse rate functions are shallower than the slopes of the T level vs. pulse rate functions. This ends up in a larger dynamic range at low pulse rates compared to that at high pulse rates [36]. The sensitivity setting determines when the AGC will start acting and is aligned to C-level stimulation [37].

Another source of discrepancy in the research studies is represented by the range of stimulation rates employed in different CI: it varies extensively from low (<500 pps/ch) to moderate (500–1000 pps/ch) to high (>1000 pps/ch) [37]. Comparing the methods used in this study to the others reported in the literature in which the beneficial effects of ASC was very evident, we applied higher stimulation rates and lower C levels.

As already mentioned, ASC works like an “automatic input volume control compressor”, modifying the T and C levels, as reported in Figure 1. Furthermore, the ASC is considered a broadband strategy, since the manipulation of microphone sensitivity affects the entire possible spectral range from 188 to 7938 Hz [28]. Several studies have investigated the effect of increasing the compression ratio and shortening the compression time constants on subjectively perceived sound quality, but the parameter of compression channel bandwidth (or number of compression channels) has not received much attention in the literature [38]. Therefore, mapping information (e.g., T/C levels, IDR/IID, number of active electrodes and rate of stimulation) was generally not cited in initial studies and shows large variations in more recent reports might significantly affect the results. In the present study, C levels were set at a lower level and at a higher rate of stimulation than those of other previous studies; thus, the addition of ASC might have led to a significant reduction of loudness as described by our patients. This, in turn, would explain the significant hearing threshold decline that negatively affects the CI recipient’s clinical performance; a similar effect is observed when a microphone fails, causing a persistent reduction in the sound processor sensitivity.

Objective measures for detecting C levels, such as those based on the stapedial reflexes [39], might provide more uniform data to evaluate the effect of these pre-processing compressions.

## 5. Conclusions

Our data indicate that word recognition performances in noise of experienced CI recipients were significantly better in the ADRO-alone than in the ADRO + ASC condition (*p* = 0.03), both with objective measures and by subjective reports. The apparent contrast with other reports in the literature may lie in the different parameters of the electrical stimulation in the different trials. 

Every CI recipient differs in T and C levels; similarly, each patient selects different preferred microphone sensitivity, volume control, and noise-reduction settings. 

We wish to stress the importance of reporting all these settings’ data e.g., T/C levels, IDR/ IID, number of active electrodes and rate of stimulation in order to compare the results obtained by different research groups studying Smartsound^®^ (Cochlear Limited, Sydney, Australia) technologies.

## 6. Summary of Evidence

1. Among this study’s subjects, 83.34% of patients performed significantly better with the ADRO alone rather than with ADRO + ASC.
2. The ASC is more useful when the change in gain is needed for larger intensity levels and longer duration.
3. The sensitivity setting determines when the AGC will start acting and is aligned to C-level stimulation. Mapping parameters (e.g., T/C levels, IDR/ IID, number of active electrodes and rate of stimulation) might significantly affect the results.

## Figures and Tables

**Table 1:** CI recipients demographic informations.

Participants	Age at Test	Sex	Device Configuration	Implanted Ear (Left(Right/Both)	Type of Hearing Loss	Age at 1st CI (Years of CI Use)	Age at 2nd CI	Interaural Equivalence	Internal Device
1	16	M	Bilateral	B	congenital	3	12	Yes	CI24RE; CI24RE
2	13	M	Bimodal	L	congenital	2			CI24RE
3	13	M	Bilateral	B	congenital	2	11	No	CI24RE; CI512
4	10	F	Bilateral	B	congenital	1	8	Yes	CI24RE; CI24RE
5	30	F	Bilateral	B	congenital	21	8	No	CI24RE; CI512
6	12	F	Bilateral	B	congenital	1	10	Yes	CI24RE; CI24RE
7	12	F	Bilateral	B	acquired	8	3	Yes	CI24RE; CI24RE
8	12	M	Bilateral	B	congenital	2	9	yes	CI24RE; CI24RE
9	47	M	Bilateral	B	acquired	38	9	Yes	CI24RE; CI512
10	8	M	Bilateral	B	congenital	1	7	Yes	CI24RE; CI24RE
11	8	M	Bilateral	B	congenital	1	7	yes	CI24RE; CI24RE
12	31	F	Bilateral	B	congenital	18	13	No	CI24RE; CI24RE
13	18	F	Bimodal	R	acquired	16			CI512
14	14	M	Bilateral	B	congenital	4	10	yes	CI24RE; CI24RE
15	14	F	Bilateral	B	congenital	2	12	No	CI24RE; CI512
16	14	M	Bilateral	B	congenital	1	13	Yes	CI24RE; CI512
17	10	M	Bilateral	B	congenital	1	9	yes	CI24RE; CI24RE
18	36	F	Bilateral	B	acquired	26	9	No	CI24RE; CI24RE

M = males; F = female; R = right ear; L: left ear; B = both ear; CI24RE: Receiver-Stimulator Contour Advance.

## Data Availability

The data presented in this study are available on request from the corresponding author. The data are not publicly available due to privacy restrictions.
